# Enhanced humoural and cellular immune responses to influenza H7N9 antigen HA1–2 fused with flagellin in chickens

**DOI:** 10.1186/s12917-017-1106-4

**Published:** 2017-06-21

**Authors:** Li Song, Dan Xiong, Maozhi Hu, Xilong Kang, Zhiming Pan, Xinan Jiao

**Affiliations:** 1 Jiangsu Co-innovation Center for Prevention and Control of Important Animal Infectious Diseases and Zoonoses, Yangzhou, Jiangsu 225009 China; 2 Jiangsu Key Laboratory of Zoonosis, Yangzhou University, 48 East Wenhui Road, Yangzhou, Jiangsu 225009 China; 3 Key Laboratory of Prevention and Control of Biological Hazard Factors (Animal Origin) for Agrifood Safety and Quality, Ministry of Agriculture of China, Yangzhou University, Yangzhou, Jiangsu China; 4 Joint International Research Laboratory of Agriculture and Agri-product Safety of the Ministry of Education, Yangzhou, Jiangsu China

**Keywords:** Avian influenza A (H7N9) virus, Haemagglutinin globular head, Flagellin, Subunit vaccine, Chicken

## Abstract

**Background:**

Sudden increases in the number of human A (H7N9) cases reported during December and January have been observed in previous years. Most reported infection cases are due to prior exposure to live poultry or potentially contaminated environments. Low pathogenicity of influenza A (H7N9) virus in avian species complicates timely discovery of infected birds. Therefore, there is a pressing need to develop safe and effective anti-H7N9 vaccines for poultry to reduce the risk of human infection and prevent the emergence of novel mutated strains. In addition to a good antigen, an effective vaccine also requires an appropriate adjuvant to enhance its immunogenicity. Previously, we generated an H7N9 influenza recombinant subunit vaccine (HA1–2-fliC), in which haemagglutinin globular head domain (HA1–2) was fused with flagellin (fliC), a potent TLR5 ligand, and demonstrated that HA1–2-fliC elicited effective HA1–2-specific immune responses in mice.

**Results:**

In this study, we determined flagellin-induced expression profiles of cytokines and chemokines in different types of avian immune cells in vitro and *ex vivo*. We found that flagellin significantly increased the expression levels of CXCL inflammatory chemokines (CXCLi1 and CXCLi2) and CCL chemokines (MIP-1β and MCP-3) in avian macrophage HD11 cells. In addition, HA1–2-fliC induced significant upregulation of cytokines (IL-1β, IL-6, IL-18 and IFN-γ) and chemokines (CXCLi1, CXCLi2 and MIP-1β) in *ex vivo* splenic lymphocytes and peripheral blood mononuclear cells (PBMCs), suggesting that flagellin promoted immune responses of avian cells in vitro. We also evaluated specific humoural and cellular immune responses induced by HA1–2-fliC and found that chickens immunised intramuscularly with HA1–2-fliC showed significantly higher HA1–2-specific immunoglobulin (Ig)G titers in serum. Furthermore, HA1–2-fliC potentiated cellular immune responses, as reflected by an increase in CD4^+^ and CD8^+^ T cells and proliferation of PBMCs. Significantly higher levels of IFN-γ and IL-4 in PBMCs from chickens vaccinated with HA1–2-fliC further indicated that HA1–2-fliC promoted a balanced Th1/Th2 immune response.

**Conclusions:**

We demonstrated that the use of the flagellin as an adjuvant potentiated immunogenicity of influenza subunit vaccine HA1–2 in vitro and in vivo*.* These findings provide a basis for the development of H7N9 influenza HA1–2 subunit vaccines for chickens.

## Background

An increase in the number of human infections with H7N9 influenza virus has been reported in China since October 2016 [1]. Between January 19th and February 14th 2017, the National Health and Family Planning Commission of People’s Republic of China (NHFPC) reported a total of 304 cases of human infection with H7N9 influenza virus [2]. The majority of infected individuals were exposed to H7N9 influenza virus through the contact with infected poultry or contaminated environments. Low avian pathogenicity of the virus adds to the difficulty in identifying its international spread through infected birds [3]. Considering the increased number of human infections with H7N9 influenza virus since December 2016, Chinese government has undertaken restrictive measures, such as closure of live poultry markets, which had a severe impact on this industry.

Therefore, there is a pressing need to develop safe and effective anti-H7N9 vaccines for poultry. It would not only decrease the risk of infecting humans who are in direct contact with poultry, but also reduce the economic loss. The most important objective is to prevent recombination with other influenza viruses in chickens, such as H9N2 influenza virus. An effective vaccine not only needs a good antigen but also requires an appropriate adjuvant to enhance the immunogenicity of the antigen [4]. Previously, we have generated an H7N9 influenza recombinant subunit vaccine that contained the globular head domain (HA1–2, aa 62–284) of the protective haemagglutinin (HA) antigen fused with the potent TLR5 ligand, *Salmonella typhimurium* flagellin (fliC), resulting in the fusion protein HA1–2-fliC [5]. We have demonstrated that HA1–2-fliC elicited effective and HA1–2-specific humoural and cellular immune responses in a mouse model [6]. In our previous study, we demonstrated that chickens vaccinated with HA1–2-fliC exhibited robust antibody responses leading to a significant reduction in viral loads of throat and cloaca compared to chickens receiving only HA1–2 [7]. However, the mechanism of adjuvant effect of flagellin in poultry is not entirely clear, such as the activation of innate immune system and the immune response types (Th1 or Th2) induced by flagellin fused with HA1–2 antigen.

It is generally known that in both poultry and mammals, the activation of the innate immune system is characterised by the production of inflammatory cytokines. Cytokines are an integral part of the immune response to pathogen infection [8]. Flagellin, a major structural protein of flagella of gram-negative bacteria, is a potent inducer of cytokine and chemokine production that has shown tremendous potential as an adjuvant in various experimental settings [9–11]. Cytokines, produced by many types of cells, modulate the activity of immune cells involved in host defence and homoeostasis. Chemokines are a large family of small secreted proteins that can chemoattract leukocyte subpopulations from the blood to the sites of inflammation. More than 20 chemokines have been identified in the chicken genome [12, 13]. In chicken, the innate immune response to *S. typhimurium* infection is induced by the production of inflammatory cytokines and chemokines such as IL-1β, IFN-γ, IL-6 and CXCLi2, in vitro and in vivo [14–16].

In this study, we evaluated the consequences of avian macrophage cell line HD11 exposure to adjuvant flagellin and investigated the effects of candidate vaccine HA1–2-fliC on peripheral blood mononuclear cells (PBMCs) and splenocytes from specific pathogen-free (SPF) chicken in vitro. In particular, we examined the expression levels of inflammatory cytokines IL-1β, IL-18, IL-6 and IFN-γ, and chemokines CXCLi1, CXCLi2, MIP-1β and MCP-3. Furthermore, chickens were immunised intramuscularly with HA1–2-fliC to investigate the humoural and cellular immune responses, in particular Th1 and Th2 immune responses, with the aim of developing an effective avian vaccine candidate against H7N9 infection.

## Methods

### Experimental chickens

Two-week-old SPF White Leghorn chickens were purchased from the poultry institute, Shandong academy of agricultural science. All birds were housed in isolation kept in a room with controlled light, temperature and ventilation parameters. Pathogen-free diet and water were supplied *ad libitum*. The procedures described in this study were approved by the Committee on the Ethics of Animal Experiments of the Yangzhou University, Yangzhou, China (Approval ID: SYXK [Su] 2012–0029).

### Preparation of proteins and adjuvants

Recombinant His-tagged HA1–2 and HA1–2-fliC proteins were expressed and purified as previously described [5]. Endotoxin was removed from the proteins by using a ProteoSpin™ Endotoxin Removal Maxi Kit (Norgen, Thorold, ON, Canada) according to the manufacturer’s instructions, and the residual endotoxin level was measured using a chromogenic endpoint tachypleus amebocyte lysate assay kit (Chinese Horseshoe Crab Reagent Manufactory Co., Ltd., Xiamen, China) according to the manufacturer’s instructions. Alum adjuvant (Thermo Fisher Scientific, Rockford, IL, USA) contained an aqueous solution of aluminium hydroxide, magnesium hydroxide and inactive stabilisers. Purified HA1–2 protein was mixed with an equal volume of alum adjuvant (*v*/v) immediately before the immunisation.

### Activation of macrophages by flagellin

The activation of macrophages by flagellin was evaluated by measuring the induction of cytokines and chemokines in HD11 cells (kindly provided by Dr. Davis Andrew from the Australian Animal Health Laboratory). Cells were cultured in 24-well microtiter plates at a seeding density of 2 × 10^6^ cells/mL in Dulbecco’s Modified Eagle’s medium supplemented with 10% foetal bovine serum (FBS) and 1% penicillin-streptomycin/L-glutamine (Gibco, Carlsbad, CA, USA). The next day, cells were treated with 5 μg/mL flagellin (Invivogen, San Diego, CA, USA) or 100 ng/mL LPS (Sigma-Aldrich, St. Louis, MO, USA). After stimulation for 5 h, cells were harvested, and the expression levels of cytokines and chemokines were evaluated via quantitative real-time PCR (qRT-PCR).

### Preparation and stimulation of chicken PBMCs and splenocytes

PBMCs and splenocytes were prepared from 30-day-old SPF chickens. PBMCs were isolated from peripheral blood using Ficoll-Hypaque (Sigma) density sedimentation. Splenic lymphocytes were obtained from the spleens of birds via density gradient centrifugation by using Lymphoprep (specific gravity 1.077; Sigma) according to the manufacturer’s instructions. PBMCs and splenocytes were suspended and cultured in 24-well microtiter plates filled with complete Roswell Park Memorial Institute 1640 medium containing 10% FBS and 1% penicillin-streptomycin/L-glutamine (Gibco, Carlsbad, CA, USA) at a final concentration of 2 × 10^6^ cells/mL. Cells were treated with 3 μg/mL HA1–2 or 10 μg/ml HA1–2-fliC (containing 3 μg HA1–2 according to the molecular weight). After incubation for 12 h, cells were harvested for RNA extraction. Cytokine and chemokine production by cells was evaluated via qRT-PCR.

### RNA extraction and RT-PCR quantification of cytokines and chemokines

Total mRNA preparations of HD11 cells, PBMCs and splenic lymphocytes were obtained by using a total RNeasy Mini kit (Qiagen, Hilden, Germany), and cDNA was synthesised from mRNA using a PrimeScrip RT reagent Kit (TaKaRa, Dalian, China) according to the manufacturer’s instructions. To determine cytokine and chemokine mRNA levels, 2 μL of diluted cDNA (40 ng/μL) was amplified in a 20-μL reaction mixture containing 10 μL of 2 × SYBR Premix Ex Taq II (Takara) and 0.6 μL of forward and reverse specific primers (10 μM) by using an ABI 7500 instrument (Applied Biosystems, Foster, CA, USA). Sequences of primers used for qRT-PCR are shown in Table 1. Data were calculated using the 2^-ΔΔCT^ approach (n-fold change compared to the control group) and reported as values normalised to the expression level of a housekeeping gene (β-actin).

**Table 1:** Sequences of primers used for quantitative real-time PCR

Gene	Primer sequences (5′-3′)	Product size (bp)	Accession no.
IFN-γ	F: agccgcacatcaaacacata	203	AJ634956.1
	R: ttggctccttttccttttga		
IL-18	F: agagcatgggaaaatggttg	168	AJ277865.1
	R: tcttcctcaaaggccaagaa		
IL-1β	F: tgggcatcaagggctaca	244	Y15006
	R: tcgggttggttggtgatg		
IL-4	F: gagaggtttcctgcgtcaag	154	AJ621249.1
	R: tgacgcatgttgaggaagag		
IL-6	F: ctcctcgccaatctgaagtc	164	AJ309540.1
	R: ggattgtgcccgaactaaaa		
CXCLi1	F: tatggctcaagcacgttcag	150	AF277660
	R: tgcaaaagcgcttacatgac		
CXCLi2	F: gcttgctaggggaaatgaag	136	AJ009800
	R: ggaattaccagtttgctgctg		
MIP-1β	F: cctcctgctgcttcacctac	158	EF197906
	R: tccaaaatgcagaggtttcc		
MCP-3	F: ctgctgcttctcctatgttcaacg	126	FR874033
	R: acacatatctccctccctttcttg		
β-actin	F: atgaagcccagagcaaaaga	223	L08165.1
	R: ggggtgttgaaggtctcaaa		

### Chicken vaccination and sample collection

A total of 32 2-week-old SPF chickens were randomly divided into four immunisation groups (*n* = 8): phosphate-buffered saline (PBS) control, 10 μg HA1–2, 30 μg HA1–2-fliC (containing 10 μg HA1–2 according to molecular weight) or 10 μg HA1–2 mixed with equal volume of alum adjuvant (*v*/v) as positive control. In each group, birds were immunised intramuscularly (i.m.) in the breast muscle with a final volume of 200 μL/dose/chicken. Identical formulations, according to the immunisation group, were given on days 0, 14 and 21.

Blood samples were collected from all chickens from the wing vein at 12 days after the second and third immunisations. Samples of serum separated from whole blood were analysed by enzyme-linked immunosorbent assay (ELISA) to determine HA1–2-specific IgG titres. At 14 days after the last vaccination, chickens were sacrificed to detect cell proliferation and numbers of CD4^+^ and CD8^+^ T lymphocytes among activated PBMCs, as well as expression levels of IFN-γ and IL-4 cytokines by splenocytes stimulated with 10 μg/mL HA1–2.

### ELISA

Serum titres of antigen-specific IgG were determined via indirect ELISA as previously described [5]. Briefly, ELISA plates were coated overnight with 1.5 μg/mL glutathione S-transferase-tagged HA1–2 antigen in 50 mM carbonate buffer (pH 9.6) at 4 °C and blocked for 2 h at 37 °C with a blocking buffer consisting of 1% bovine serum albumin in PBS with 0.5% Tween-20. Plates were washed and then samples were added and serially diluted with a dilution buffer within the plate followed by the incubation for 2 h at 37 °C. Horseradish peroxidase-conjugated anti-chicken IgY (IgG; 1:10,000; Sigma) was added as the secondary antibody and incubated for 1 h at 37 °C, with 3, 3′, 5, 5′-tetramethylbenzidine (TMB) used as the substrate to estimate enzymatic activity. The reaction was stopped with 2 M H_2_SO_4_, and the absorbance was measured at 450 nm using a microplate reader (Bio-Tek, Winooski, VT, USA).

### Cell proliferation

PBMCs were obtained from chickens after second and third immunisations as described above, and PBMC proliferation was evaluated using a commercially available ELISA-BrdU kit (Roche Diagnostics, Tokyo, Japan) according to the manufacturer’s protocol. Briefly, PBMC suspensions (100 μL/well or 2 × 10^5^ cells/well) were pre-treated with 10 μg/mL ConA in triplicate in a 96-well plate or left untreated and incubated at 37 °C in a 5% CO_2_ incubator for 48 h. BrdU labelling reagent was then added at 10 μL/well to the final concentration of 10 mM. After 12 h, cells were harvested via centrifugation, and the plate was dried at 60 °C for 1 h. BrdU-labelled DNA in the cells was fixed and denatured by the incubation with a FixDenat solution for 30 min at room temperature. After washing, DNA was stained with a peroxidase-conjugated anti-BrdU antibody for 90 min at room temperature. After additional washes, TMB substrate solution was added, and the mixtures were incubated at room temperature until the appropriate colour developed. The reaction was stopped by adding 1 M H_2_SO_4_ solution. The optical density of each sample (OD450/690) was measured at a wavelength of 450 nm with background subtraction at 690 nm by using an ELISA plate reader. Stimulation index (SI) was calculated using the following equation: SI = (OD450 − OD690 of antigen-treated cells)/(OD450 − OD690 of untreated cells).

### Flow cytometry

PBMCs were obtained from chickens on the 14th day after the third immunisation as described above for preparations of CD4^+^ and CD8^+^ T cell populations. Lymphocytes (2 × 10^6^ cells/mL) were washed twice in PBS, resuspended in PBS and incubated for 30 min at room temperature with the fluorescently labelled monoclonal antibodies anti-CD4-PE, anti-CD8a-APC and anti-CD3-FITC (BD Pharmingen) according to the manufacturer’s technical bulletin. Then, preparations were washed three times with PBS. Labelled cells were analysed using a FACS Aria flow cytometer (BD Biosciences) with FACSDiva software (BD Biosciences).

### Statistical analysis

All results are expressed as the mean ± standard error of the mean, unless otherwise stated. Statistical significance of differences between two groups was analysed by the unpaired Student’s *t*-test using Prism 5.0 (GraphPad Software, Inc., San Diego, CA, USA). Differences were considered statistically significant if *P* < 0.05.

## Results

### Activation of avian macrophages by flagellin in vitro

**Fig. 1:**
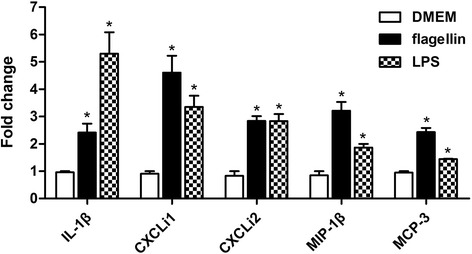
Cytokine gene expression in HD11 cells. Cells (2 × 10^6^ cells/mL) were treated with 5 μg/mL flagellin, 100 ng/mL LPS (positive control) or left untreated (DMEM) for 5 h, and mRNA levels were measured via qRT-PCR. Data are presented as the mean ± standard error of the mean. Statistical significance of differences is illustrated as follows: *P < 0.05

Macrophage activation induced by flagellin was evaluated by measuring the expression levels of inflammatory cytokines and chemokines in HD11 cells treated with 5 μg/mL flagellin. We observed that exposure to flagellin significantly upregulated (*P* < 0.05) the expression level of the pro-inflammatory cytokine IL-1β (2.4 fold) and inflammatory chemokines CXCLi1 (4.6 fold), CXCLi2 (2.8 fold), MIP-1β (3.2 fold) and MCP-3 (2.4 fold) compared to their levels in untreated group (Fig. 1).

### Activation of chicken immune cells by HA1–2-fliC *ex vivo*

**Fig. 2:**
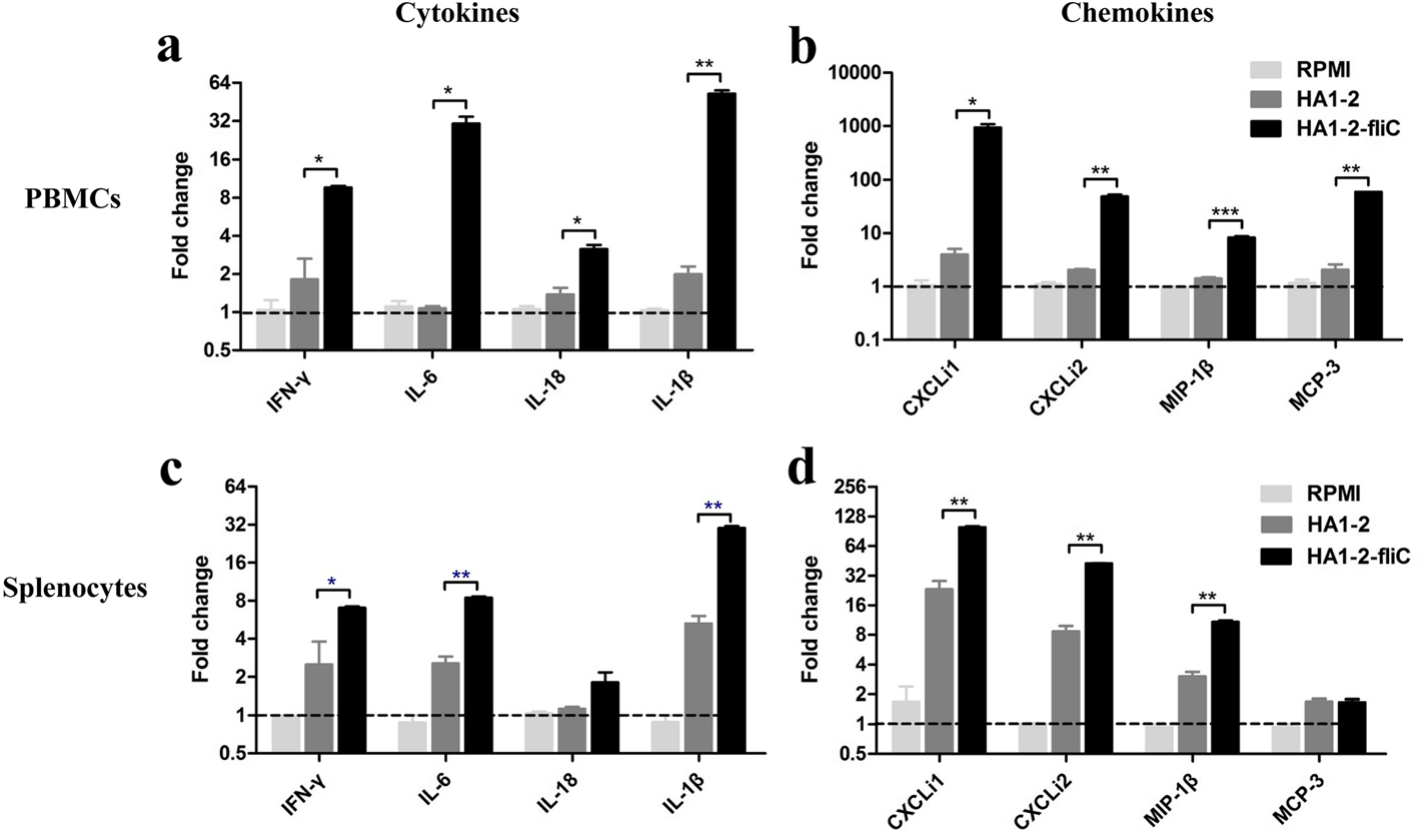
Cytokine and chemokine mRNA levels in PBMCs and splenic lymphocytes. Splenic lymphocytes and PBMCs isolated from mature specific pathogen-free chickens were treated with HA1–2 and HA1–2-fliC proteins for 12 h in vitro. Expression levels of cytokines and chemokines in PBMCs (**a**, **b**) and splenic lymphocytes (**c**, **d**) were measured by qRT-PCR. Data are presented as the mean ± standard error of the mean. Statistical significance of differences is illustrated as follows: *P < 0.05, **P < 0.01, ***P < 0.001

PBMCs and splenocytes were isolated from chickens and treated with HA1–2 and HA1–2-fliC. We found that among the cytokines investigated (IL-1β, IL-6, IL-18, IFN-γ), fold change of mRNA expression levels of pro-inflammatory IL-1β (52.5 fold, *P* < 0.01; 30.1 fold, *P* < 0.01), IL-6 (30.5 fold, *P* < 0.05; 8.4 fold, *P* < 0.01) and IFN-γ (9.6 fold, *P* < 0.05; 7.0 fold, *P* < 0.05) were significantly higher in HA1–2-fliC-treated PBMCs and splenocytes, respectively, compared to their levels in HA1–2-treated group, whereas IL-18 mRNA expression level was significantly higher (3.1 fold, *P* < 0.05) only in PBMCs (Fig. 2a and c). With regard to chemokine changes, significantly higher mRNA expression levels of CXCLi1 (941.4 fold, *P* < 0.05; 99.1 fold, *P* < 0.01), CXCLi2 (48.9 fold, *P* < 0.01; 42.5 fold, *P* < 0.01) and MIP-1β (8.3 fold, *P* < 0.001; 10.8 fold, *P* < 0.01) were detected in PBMCs and splenocytes treated with HA1–2-fliC than in those treated with HA1–2. MCP-3 expression level was significantly higher (59.6 fold, *P* < 0.01) only in PBMCs (Fig. 2b and d).

### HA1–2-fliC promotes antibody immune responses

**Fig. 3:**
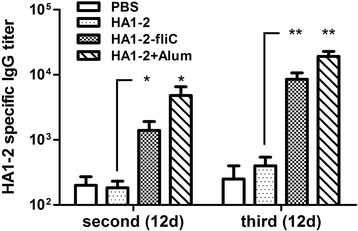
Serum titres of HA1–2-specific IgG. Specific pathogen-free chickens (*n* = 8) were vaccinated intramuscularly with three doses of candidate vaccines. Chickens were subjected to bleeding in 12 days after the second and third immunisations. Serum IgG titres were measured via enzyme-linked immunosorbent assay. Data are presented as the mean ± standard error of the mean. Statistical significance of differences is illustrated as follows: *P < 0.05, **P < 0.01

To determine antigen-specific immune responses to immunisation, serum samples from immunised chickens were collected on day 12 after the second and third immunisations and tested for titres of HA1–2-specific IgG (Fig. 3). HA1–2-fliC induced significantly higher HA1–2-specific IgG titres than did HA1–2 alone after both the second (1400 vs. 183, *P* < 0.05) and third (8533 vs. 400, *P* < 0.01) vaccinations.

### HA1–2-fliC enhances cellular immune responses

**Fig. 4:**
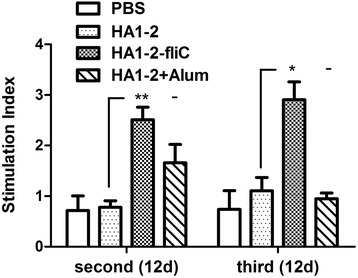
Proliferation of PBMCs. PBMCs were isolated from chickens vaccinated intramuscularly at 2 weeks after second and third inoculations, and the cellular immune response was assessed by monitoring cell proliferation. Stimulation index (SI) was calculated based on cell proliferation, as determined using the ELISA-BrdU assay and the following equation: SI = (OD450 − OD690 of antigen-treated cells)/(OD450 − OD690 of untreated cells). Data are presented as the mean ± standard error of the mean. Statistical significance of differences is illustrated as follows: *P < 0.05, **P < 0.01

At 12 days after the third immunisation, chicken PBMCs were prepared, and cellular immune responses were assessed by monitoring lymphocyte proliferation with the BrdU assay. As shown in Fig. 4, the values of SI of lymphocytes in HA1–2-fliC group after the second (2.5 on average, *P* < 0.01) and third (2.9 on average, *P* < 0.05) vaccinations were significantly higher than those in HA1–2 group.

**Fig. 5:**
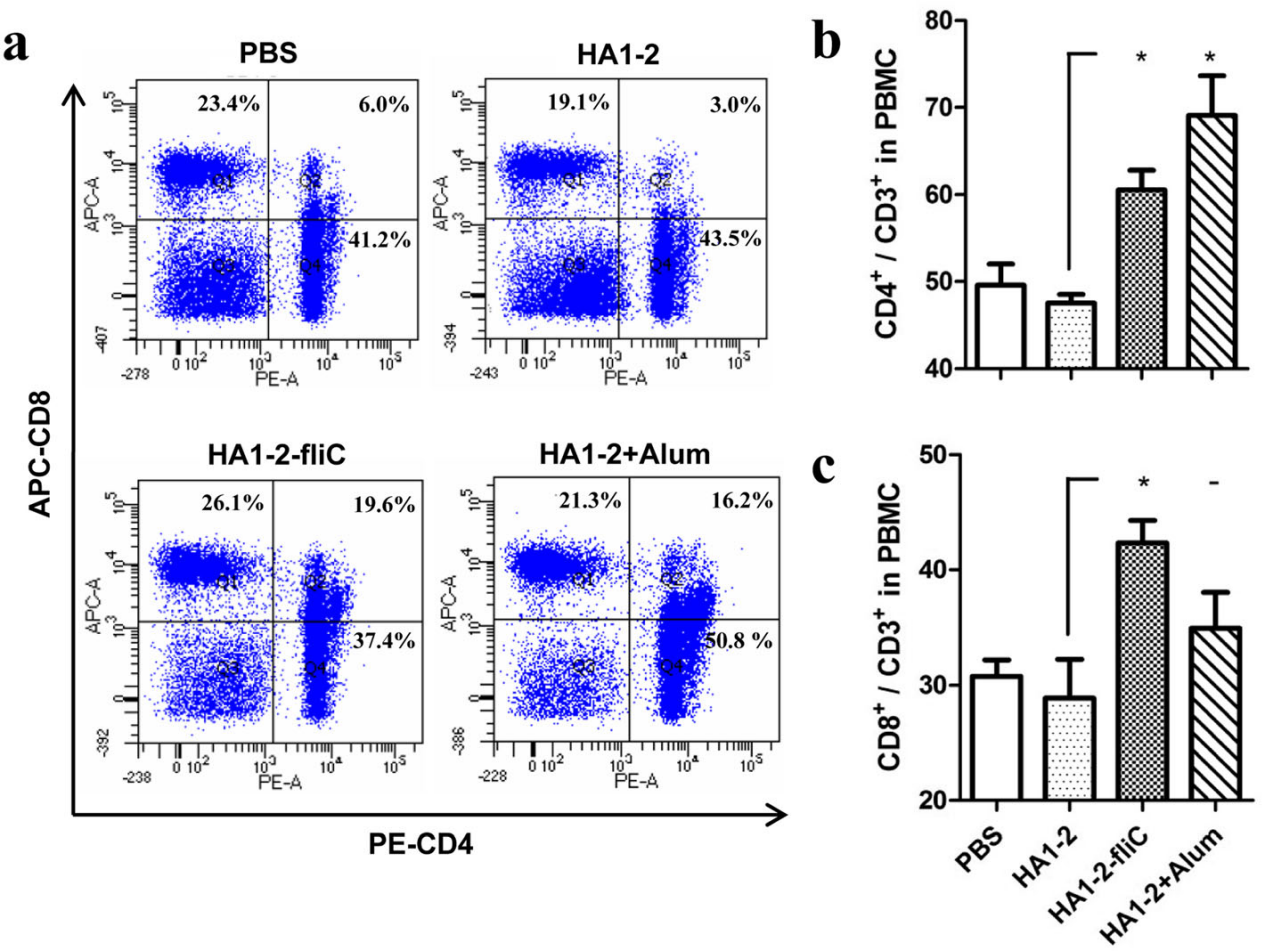
Flow cytometry analysis of PBMCs. PBMCs were isolated from chickens vaccinated intramuscularly at 2 weeks after the third inoculation, and the cellular immune response was assessed by monitoring T-cell subsets. **a**: Representative images showing distribution of CD4^+^ T cells (CD3^+^ CD4^+^) and CD8^+^ T cells (CD3^+^ CD8^+^). **b**: CD4^+^ T cell percentages in PBMCs in different treatment groups. **c**: CD8^+^ T cell percentages in PBMCs in different treatment groups. Data are presented as the mean ± standard error of the mean. Statistical significance of differences is illustrated as follows: *P < 0.05

To further determine parameters of T lymphocyte activation, T-cell subset percentages were analysed via flow cytometry. At 12 days after the third vaccination, the percentages of CD4^+^ T cells (CD3^+^ CD4^+^) and CD8^+^ T cells (CD3^+^ CD8^+^) in peripheral blood lymphocyte populations were significantly higher in chickens immunised with HA1–2-fliC (60.55%, *P* < 0.05 and 42.35%, *P* < 0.05, respectively) compared with those in chickens immunised with HA1–2 alone (47.5 and 28.8, respectively; Fig. 5). These data suggest that HA1–2-fliC stimulated the activation of both CD4^+^ and CD8^+^ T cells.

### HA1–2-fliC augments both Th1- and Th2-type immune responses

**Fig. 6:**
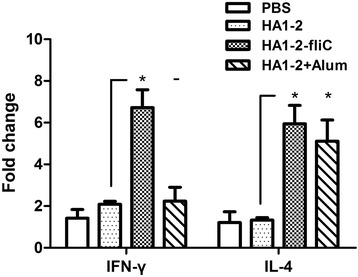
IFN-γ and IL-4 expression levels in splenocytes of SPF chickens. Spleens were isolated from chickens (*n* = 8) vaccinated intramuscularly at two weeks after the third inoculation, and the levels of IFN-γ and IL-4 in the spleens at 5 h post-stimulation were determined by qRT-PCR. Data are presented as the mean ± standard error of the mean. Statistical significance of differences is illustrated as follows: *P < 0.05

Levels of the cytokines IFN-γ and IL-4, associated with Th1-type and Th2-type immune responses, respectively, were determined to evaluate the influence of fliC adjuvant on H7N9 influenza subunit vaccine in SPF chickens. We found that mRNA levels of IFN-γ and IL-4 from the spleens of chickens vaccinated with HA1–2-fliC were significantly higher (6.7 fold and 5.9 fold, respectively, *P* < 0.05) at 12 days after vaccination than IFN-γ and IL-4 levels in the spleens of HA1–2 vaccinated chickens (Fig. 6).

## Discussion

In chicken, TLR5 has been reported to be expressed in various organs [17]. In particular, TLR5 expression has been detected in many immune cells of chicken, such as heterophils, monocytes, Langerhans cells, NK cells, as well as in T and B cells of the adaptive immune system [18–20]. It has been demonstrated previously that the flagellar protein flagellin of flagellated bacteria, such as *S. enterica* serovar Typhimurium, can activate the immune system of the host [21, 22]. In contrast, the absence of flagellin, e.g., in the nonflagellated bacterium *S. enterica* serovar Gallinarum, leads to lesser invasiveness and lower levels of cytokine and chemokine induction in the host [21]. In this context, we evaluated the priming effects of flagellin and fusion protein HA1–2-fliC in vitro on immune responses in avian macrophage cell line HD11, as well as in PBMCs and splenocytes obtained from SPF chickens, by examining the expression levels of cytokines and chemokines.

Chemokines are a large family of small secreted proteins that can chemoattract leukocyte subpopulations from the blood to the sites of inflammation. It has been proposed that chemokines should be referred to as CXCLi, and the “i” denoting inflammatory function [12]. In this study, we found that mRNA expression levels of inflammatory chemokines CXCLi1, CXCLi2, MIP-1β and MCP-3 were strongly induced by flagellin in avian macrophage HD-11 cells to the levels comparable with those obtained after exposure to LPS (Fig. 1). Because LPS is known to induce a wide range of cytokines in mammalian macrophages [23], it was used to stimulate chicken macrophage HD-11 cells as positive control.

Recent studies aimed at exploring the potential of flagellin as an adjuvant have reported induction of mixed Th1 and Th2 immune responses in chicken cells [24, 25]. In this study, in PBMCs and splenic lymphocytes isolated from mature SPF chickens, mRNA levels of the Th1 cytokines IFN-γ and IL-18 were significantly higher in cells stimulated with HA1–2-fliC than in those treated with HA1–2 (Fig. 2a and c). Furthermore, Th2 cytokine IL-6 levels were also significantly higher in PBMCs (Fig. 2a). In addition, higher levels of IL-1β mRNA were also detected in PBMCs and splenocytes. It has been demonstrated previously that rapid and significant elevations in IL-1β and CXCLi2 mRNA expression were observed upon immunisation with recombinant *Salmonella* strains in the cecum and spleen [26].

TLR5-flagellin-mediated upregulation of IL-1β, IL-6 and CXCLi2 mRNA expression levels have been detected in cells infected with *S. enterica* serovar Enteritidis [22]. In addition, we found that expression of MIP family chemokines significantly increased in HD11 cells, PBMCs and splenocytes. These observations were in line with previous reports about early expression of MIP family chemokines in the spleen of 1-week-old chicken induced by the infection with *Salmonella* serovar Typhimurium [16]. MIP-1 is expressed in activated macrophages, lymphocytes and fibroblasts, and it renders cells chemoattractive towards T cells [27]. All these findings suggest that HA1–2-fliC has the capacity, at least in vitro, to promote immune responses in avian cells.

Many in vivo investigations have been undertaken to follow up promising results of various in vitro studies of the immunogenicity of purified or recombinant flagellin in chicken immune cells [28, 29]. In this regard, we explored whether in vivo administered flagellin adjuvanted subunit vaccine enhanced antigen specific immune responses. We found that HA1–2-specific serum IgG titres induced by HA1–2-fliC were significantly higher than those induced by HA1–2 alone, indicating high potency of fliC as an adjuvant for H7N9 influenza subunit vaccines (Fig. 3). Similarly, Chaung et al. [29] investigated adjuvant effects of the monomeric and polymeric forms of *Salmonella* flagellin in chickens immunised intramuscularly with formalin-inactivated avian influenza virus H5N2 vaccines and demonstrated that IgG titres were significantly higher when animals were vaccinated with the vaccine that contained flagellin adjuvant.

The ideal vaccine stimulates not only serum IgG production but also the cellular response [30]. T cells play an important role in protection against various strains of influenza virus [31]. We evaluated PBMC proliferation and cytokine secretion by splenocytes following HA1–2 stimulation. As expected, PBMC proliferation was significantly higher in HA1–2-fliC group (Fig. 4), consistent with the results of a previous study that reported significantly higher proliferation of PMBCs from chickens vaccinated with mFliC or pFliC-adjuvanted vaccines [29]. Furthermore, IFN-γ and IL-4 mRNA levels were significantly higher in splenocytes from HA1–2-fliC-treated chickens than in chickens administered with HA1–2 alone, suggesting that antigen-specific lymphocytes were strongly activated in the presence of the adjuvant.

In our previous study on a chicken model, we detected the antibody immune responses induced by HA1–2-fliC [7], whereas cellular immune responses are more important for protecting the host against influenza virus. Here, we focused on the detection of the activation of immune cells by qRT-PCR and flow cytometry. Th1 and Th2 CD4^+^ T cells are primarily associated with cellular immune responses and antibody production, respectively [32]. In our experiments, HA1–2-fliC vaccine induced expression of significantly higher levels of IFN-γ and IL-4 (Fig. 6). Moreover, the percentages of CD4^+^ T cells and CD8^+^ T cells in the peripheral blood lymphocyte populations were significantly higher in chickens immunised with HA1–2-fliC than with HA1–2 only (Fig. 5), indicating that chickens vaccinated with HA1–2-fliC exhibited a balanced Th1/Th2 immune response. In contrast, another study reported that co-delivery of fliC with an antigen induced predominantly a Th2 response in mice [33], indicating that distinct immune responses may be induced by flagellin in different animal models.

In chickens, many combinations have been tried to establish the best pattern of cytokine production and induction of the type of immune responses. For example, a combination of CpG oligodeoxynucleotides and polyinosinic-polycytidylic acid synergistically stimulated pro-inflammatory immune response in chicken monocytes by activating nitric oxide production and by inducing expression of iNOS and proinflammatory cytokines and chemokines [34, 35]. Flagellin has also been used in combination with other agonists. In a previous study, induced crosstalk between TLR5 and TLR9 in human PBMCs resulted in a more robust production of IL-10 and IFN-γ, but inhibited the expression of IL-12 [36]. Therefore, in such cases, a novel combination of TLR ligands can be tried to achieve more effective and selective immune responses (Th1 or Th2) in chickens.

In addition, we have demonstrated that the HA1–2-fliC protein can be successfully and efficiently expressed using an *E. coli* prokaryotic system [5], which could lead to the potential development of industrial large-scale production of vaccines. The idea has been confirmed in some studies. The *E. coli*-based VLPs vaccine against foot-and-mouth disease virus infections was produced in large scale by fermentation at 10,000 mL scale [37], and a new rVP2 subunit vaccine expressed by *E. coli* against infectious bursal disease was fermented in 20,000 mL culture medium [38]. These researches will make it possible for the subunit vaccines to be produced commercially and used in poultry industry in large scale.

## Conclusions

We evaluated the effects of flagellin in vitro on innate immune responses using avian macrophage cell line HD11, as well as the activity of HA1–2-fliC by PBMCs and splenocytes obtained from SPF chickens. Significantly higher expression levels of inflammatory cytokines and chemokines were observed after exposure to the flagellin adjuvanted vaccine HA1–2-fliC than that to HA1–2 alone, indicating that HA1–2-fliC has the capacity to promote innate immune activation in avian cells. The results showed that the HA1–2-fliC could elicit significantly stronger humoural and cellular immune responses in chickens with balanced Th1 and Th2 immune responses. These findings provide the foundation for the development of a promising HA1–2 subunit vaccine for H7N9 influenza in chickens.

## Abbreviations

CXCLi: chemokine (C-X-C motif) ligand inflammatory
ELISA: enzyme-linked immunosorbent assay
FBS: foetal bovine serum
HA: haemagglutinin
MCP-3: monocyte chemoattractant protein-3
MIP-1β: macrophage inflammatory protein-1β
NHFPC: National Health and Family Planning Commission of People’s Republic of China
PBMCs: peripheral blood mononuclear cells
PBS: phosphate buffered saline
qRT-PCR: quantitative real-time PCR
SI: stimulation index
SPF: specific pathogen-free
TMB: 3, 3′, 5, 5′-tetramethylbenzidine
